# Raf kinase inhibitor protein: lessons of a better way for β‐adrenergic receptor activation in the heart

**DOI:** 10.1113/JP274064

**Published:** 2017-05-23

**Authors:** Kristina Lorenz, Marsha Rich Rosner, Theresa Brand, Joachim P Schmitt

**Affiliations:** ^1^ Comprehensive Heart Failure CenterUniversity of WürzburgVersbacher Straße 997078WürzburgGermany; ^2^ West German Heart and Vascular Center EssenUniversity Hospital EssenHufelandstraße 5545147EssenGermany; ^3^ Leibniz‐Institut für Analytische Wissenschaften – ISAS – e.V.Bunsen‐Kirchhoff‐Straße 1144139DortmundGermany; ^4^ Institute of Pharmacology and ToxicologyUniversity of WürzburgVersbacher Straße 997078WürzburgGermany; ^5^ Institute of Pharmacology and Clinical PharmacologyDüsseldorf University HospitalUniverstitätsstraße 140225DüsseldorfGermany; ^6^ Cardiovascular Research Institute Düsseldorf (CARID)Heinrich‐Heine‐UniversityUniverstitätsstraße 140225DüsseldorfGermany; ^7^ Ben May Department for Cancer ResearchUniversity of ChicagoChicagoIL 60637USA

**Keywords:** beta‐adrenergic receptors, heart failure, RKIP

## Abstract

Stimulation of β‐adrenergic receptors (βARs) provides the most efficient physiological mechanism to enhance contraction and relaxation of the heart. Activation of βARs allows rapid enhancement of myocardial function in order to fuel the muscles for running and fighting in a fight‐or‐flight response. Likewise, βARs become activated during cardiovascular disease in an attempt to counteract the restrictions of cardiac output. However, long‐term stimulation of βARs increases the likelihood of cardiac arrhythmias, adverse ventricular remodelling, decline of cardiac performance and premature death, thereby limiting the use of βAR agonists in the treatment of heart failure. Recently the endogenous Raf kinase inhibitor protein (RKIP) was found to activate βAR signalling of the heart without adverse effects. This review will summarize the current knowledge on RKIP‐driven compared to receptor‐mediated signalling in cardiomyocytes. Emphasis is given to the differential effects of RKIP on β_1_‐ and β_2_‐ARs and their downstream targets, the regulation of myocyte calcium cycling and myofilament activity.

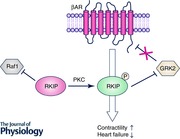

**Abbreviations**
AAV: adeno‐associated virus
AC6: adenylyl cyclase 6
βAR: β‐adrenergic receptor
βARKct: β‐adrenergic receptor kinase, C‐terminus
CaMKII: Ca^2+^/calmodulin‐dependent protein kinase II
cAMP: cyclic adenosine monophosphate
cMyBPC: cardiac myosin binding protein C
Epac: exchange protein directly activated by cAMP
ERK1/2: extracellular signal‐regulated protein kinases 1 and 2
GPCR: G‐protein‐coupled receptor
GRK: G‐protein‐coupled receptor kinase
G_i_: inhibitory G‐protein
G_s_: stimulatory G‐protein
KO: knock‐out
LTCC: L‐type Ca^2+^ channel
MEK: mitogen‐activated protein kinase kinase
NCX: sodium–calcium exchanger
PDEIII: phosphodiesterase III
PEBP: phosphatidylethanolamine‐binding protein
PKA: protein kinase A
PKC: protein kinase C
PLN: phospholamban
PMCA: sarcolemmal Ca^2+^‐ATPase
RKIP: Raf kinase inhibitor protein
RyR2: ryanodine receptor 2
S100A1: S100 calcium binding protein A1
SERCA2a: sarco‐/endoplasmic reticulum Ca^2+^‐ATPase
SR: sarcoplasmic reticulum
TnI: troponin I

## Introduction

Heart failure occurs if cardiac output is reduced to an extent that it cannot meet the body's needs. It represents one of the leading causes of morbidity and mortality in developed countries and results from the loss and/or dysfunction of cardiomyocytes due to literally any insult to the heart, most frequently chronic arterial hypertension, myocardial infarction, aortic stenosis or infectious diseases. The activation of the sympathetic nervous system via β‐adrenergic receptors (βARs) is the most important compensatory mechanism in heart failure, acting to stabilize the hemodynamic situation by accelerating cardiac contraction and relaxation (Ponikowski *et al*. 2016).

Free calcium ions (Ca^2+^) are the critical intermediary that translates sympathetic activity into myofilament movement. Changes of beat‐to‐beat myocyte Ca^2+^ cycling are also among the hallmarks of heart failure. Further, local Ca^2+^ release events (Ca^2+^ sparks) and/or altered Ca^2+^ sensitivity of cardiomyocytes contribute to contractile dysfunction and increase the risk of cardiac arrhythmias in failing hearts as well as in diseases like inherited cardiomyopathy or early after myocardial infarction (Cho *et al*. 2016). The profound effects on cardiac function of even small modifications to elements of the βAR signalling cascade, the receptor cascade that controls Ca^2+^ handling to the myofilaments, necessitates precise regulation of the entire system.

In the treatment of heart failure, pharmacological activation of βARs is beneficial in acute situations due to its ability to rapidly increase cardiac output. However, sustained activation of βARs is detrimental to the heart; it promotes cardiomyocyte death and myocardial fibrosis and increases patient mortality (Engelhardt *et al*. 1999; Tacon *et al*. 2012; Ponikowski *et al*. 2016). A strategy that would increase cardiac output, but without the adverse effects of chronic βAR stimulation, is still lacking. This review will discuss novel therapeutic approaches aimed at selective activation of specific components of βAR signalling with a main focus on the Raf kinase inhibitor protein (RKIP). Upon phosphorylation by protein kinase C (PKC), RKIP potentiates βAR signalling through inhibition of receptor desensitization, which has proven beneficial effects on myocyte Ca^2+^ regulation and murine heart failure (Lorenz *et al*. 2003; Schmid *et al*. 2015).

## βAR signalling in the heart

Sympathetic activity is transmitted to cardiac muscle via neuronally and circulating catecholamines that predominantly activate βARs on cardiomyocytes as those are the receptor subtypes with the highest density in the ventricular myocardium. Two different βAR subtypes are expressed in the heart: β_1_‐ and β_2_‐adrenergic receptors, at a ratio of 80:20. Stimulation of cardiac βARs mediates an increase in contractile force (positive inotropy), speed of relaxation (positive lusitropy), atrioventricular conduction (positive dromotropy) and heart rate (positive chronotropy) (Bristow *et al*. 1986; Brodde 1991; Jensen *et al*. 2009). β_3_ARs are a third subtype of cardiomyocyte βAR. Their role in the heart, however, is still largely unclear. They induce distinct intracellular signalling pathways and a negative inotropic effect. Since their expression is increased in several subtypes of human cardiomyopathy, they may have a potential role in heart failure. Mice with cardiac β_3_AR overexpression showed reduced hypertrophic remodelling through nitric oxide synthase activation (Balligand 2013; Belge *et al*. 2014).

**Figure 1:**
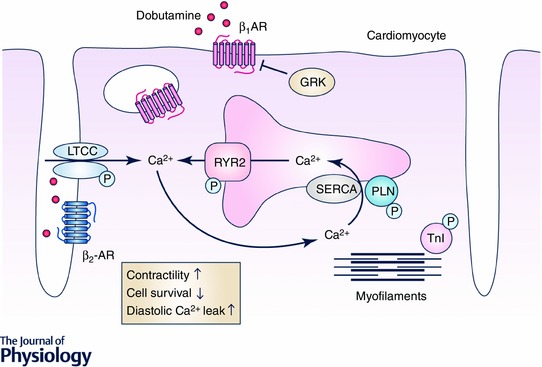
Acute dobutamine application induces positive inotropy; chronic dobutamine application deteriorates cardiac function Dobutamine activates β_1_‐ and β_2_‐adrenergic receptors (β_1_AR and β_2_AR). Activated βARs increase contractility and relaxation of cardiomyocytes via the activation of stimulatory G‐proteins (G_s_), which in turn activate protein kinase A and Ca^2+^/calmodulin‐dependent protein kinase II. These kinases increase Ca^2+^ cycling: upon phosphorylation, phospholamban (PLN) dissociates from sarco‐/endoplasmatic reticulum Ca^2+^‐ATPase (SERCA2a). This leads to increased SERCA2a‐mediated Ca^2+^ re‐uptake into the sarcoplasmatic reticulum and cardiomyocyte contractility. Phosphorylation of troponin I (TnI) decreases Ca^2+^ sensitivity and thereby increases cardiomyocyte relaxation. However, G‐protein‐coupled receptor kinase (GRK) phosphorylates activated G‐protein‐coupled receptors (GPCR) as for example β_1_AR and β_2_AR, which induces receptor desensitization and internalization. This blunts βAR signalling and the initial increase in cardiomyocyte contractility upon dobutamine application. Further, chronic βAR stimulation induces apoptosis, fibrosis and arrhythmia, in particular via hyperphosphorylation of the ryanodine receptor 2 (RyR2) and L‐type Ca^2+^ channels (LTCC), thereby leading to increased diastolic Ca^2+^ leak.

β_1_‐ and β_2_ARs couple to stimulatory G‐proteins (G_s_) that stimulate adenylyl cyclases to produce the second messenger cyclic adenosine monophosphate (cAMP), which in turn activates the cAMP‐dependent protein kinase A (PKA). In cardiomyocytes, regulators of beat‐to‐beat Ca^2+^ cycling and sarcomere proteins represent major substrates of PKA. Activation of PKA causes phosphorylation of L‐type Ca^2+^ channels (LTCCs). This increases Ca^2+^ influx; phosphorylation of phospholamban (PLN), which accelerates the reuptake of Ca^2+^ into the sarcoplasmatic reticulum (SR); phosphorylation of ryanodine receptors 2 (RyR2), which increases SR Ca^2+^ release; phosphorylation of troponin I (TnI) and of cardiac myosin binding protein C (cMyBPC), which decreases myofilament Ca^2+^ sensitivity; and phosphorylation of titin, which reduces the sarcomeric passive stiffness (Lefkowitz *et al*. 2002; Rockman *et al*. 2002; Krüger & Linke, 2006; Baker, 2014; Najafi *et al*. 2016). Taken together, these PKA‐mediated phosphorylation events enhance Ca^2+^ cycling and reduce myofilament Ca^2+^ sensitivity in cardiomyocytes, leading to the increases in force and increases in the rates of contraction and of relaxation. Myocyte Ca^2+^ is also important for the formation of the Ca^2+^–calmodulin complex, which activates Ca^2+^/calmodulin‐dependent protein kinase II (CaMKII), a kinase that also impacts on Ca^2+^ homeostasis by phosphorylation of RyR2, PLN or cMyBPC, thereby further potentiating βAR‐mediated cardiac contraction and relaxation (Fig. 1; Rockman *et al*. 2002; Maier & Bers, 2007; Lehnart *et al*. 2009; Sadayappan *et al*. 2011; Uchinoumi *et al*. 2016). In addition, βARs activate an exchange protein directly activated by cAMP (Epac). Epac1 seems to contribute to cardiac hypertrophy and is upregulated in heart failure, whereas Epac2 seems to be involved in CaMKII‐induced SR Ca^2+^ leak and arrhythmia. Despite these detrimental effects, Epac has also been reported to promote cardiomyocyte survival in heart failure (Métrich *et al*. 2008, 2009; Pereira *et al*. 2013).

Thus, βARs, as major drivers of heart rate, contractile force, speed of contraction and relaxation, play an important role in so‐called fight‐or‐flight situations or whenever cardiac output needs to be enhanced. Analogously, βAR agonists such as adrenaline, dobutamine and dopamine are used to stabilize patients in *acute* cardiac failure (Felker, 2001; Tacon *et al*. 2012; Ponikowski *et al*. 2016). However, long‐lasting application of these β‐AR agonists for several days or even weeks induces structural cardiac damage, including cardiac hypertrophy, cardiomyocyte apoptosis and interstitial fibrosis (Fig. 1; Engelhardt *et al*. 1999; O'Connor, *et al*. 1999; Felker, 2001; Tacon *et al*. 2012; Vidal *et al*. 2012; Ponikowski *et al*. 2016). As a natural defence against this damage, prolonged activation of βAR leads to receptor desensitization via phosphorylation by G‐protein‐coupled receptor kinases (GRK), thereby protecting the heart from long‐term sympathetic overdrive. The predominant GRK subtype in the heart is GRK2. Phosphorylation of βARs by GRK2 increases the affinity of the receptor for β‐arrestin, a protein that blocks G‐protein coupling upon receptor stimulation and promotes receptor internalization and degradation, rendering myocytes less responsive to agonist binding of βARs (Rockman *et al*. 2002). However, such loss of βAR function also promotes contractile decline of failing hearts. This dilemma between the need for positive inotropy of failing hearts on one side and receptor desensitization to prevent cardiac damage upon sustained βAR activation on the other side pushes for a well‐synchronized, well‐balanced and fine‐tuned way to regulate βAR signalling in the heart.

## βAR signalling in heart failure

βAR agonists augment cardiac contraction at the beginning of treatment. In contrast, sustained βAR stimulation is cardiotoxic, consistent with the finding that noradrenaline plasma levels correlate with the degree of cardiac dysfunction and mortality of heart failure patients (Thomas & Marks, 1987; Cohn *et al*. 1993; Zhang *et al*. 2013). Under conditions of increased sympathetic nervous system activation or chronic βAR agonist treatment, both βAR density at the surface of the cell membrane and the responsiveness of the remaining receptors are reduced. These molecular characteristics of failing hearts correlate well with the stage of heart failure independent of the underlying cause of the disease (Ohsuzu *et al*. 1994). The pattern of βAR subtype downregulation, however, seems to depend on the aetiology of heart failure: β_1_ARs but not β_2_ARs are downregulated in the majority of heart failure cases, but in mitral valve disease and ischaemic cardiomyopathy both βAR subtypes are affected to a similar extent (Brodde *et al*. 1986; Bristow *et al*. 1991; Steinfath *et al*. 1991, 1992).

Toxic effects mediated through βAR activation appear to originate from β_1_ARs, because cardiac overexpression of β_1_ARs in mice led to cardiac hypertrophy, interstitial fibrosis and cardiac dysfunction (Engelhardt *et al*. 1999; Zhang *et al*. 2013). Furthermore, β_1_ARs mediate pro‐apoptotic signalling through the kinases PKA and CaMKII. For example, selective inhibition of β_1_AR resulted in protection of catecholamine‐induced apoptosis in rat ventricular myocytes (Zaugg *et al*. 2000; Shizukuda & Buttrick, 2002).

β_2_ARs in contrast, have been described as cardioprotective receptors (Liggett *et al*. 1998; Siedlecka *et al*. 2008). Cardiac overexpression of β_2_ARs in mice prevented myocardial remodelling and contractile dysfunction in a genetic model of heart failure generated by G_αq_ overexpression (Dorn *et al*. 1999). However, favourable effects were achieved only at relatively low levels of β_2_AR overexpression, whereas higher expression levels turned out deleterious, suggesting that specificity of β_2_AR signalling must be preserved to achieve beneficial effects via this activation. In addition, β_2_AR overexpression of up to 60‐fold was tolerated in ageing mouse hearts without detriment for a period of at least 1 year (Liggett *et al*. 2000). Further, selective β_2_AR activation protected from stress‐induced apoptosis in isolated cardiomyocytes as well as from myocardial dysfunction and apoptosis in a rat model of heart failure (Paur *et al*. 2012) and mice lacking β_2_ARs had a higher mortality than wild‐type mice in response to chronic isoproterenol application (Patterson *et al*. 2004). Also, in human heart failure an Ile164 polymorphism in the β_2_AR, which reduces its signalling efficiency, was found to worsen patients’ prognosis (Liggett *et al*. 1998). Beneficial effects of β_2_ARs in the heart are often associated with β_2_AR coupling to inhibitory G‐proteins (G_i_). In line with this, the unfavourable outcome of the Ile164 polymorphism was suggested to result from the loss of β_2_ARs coupled to G_i_ and their protective effects on apoptosis (Chesley *et al*. 2000). On the other hand, enhanced β_2_AR–G_i_ signalling is also reported to contribute to cardiac deterioration in heart failure by further reducing cardiac contractility. Thus, several groups hypothesized that a combination of β_1_AR blockade with β_2_AR–G_s_ activation may be ideal for improving cardiac contractility without adverse effects (Ahmet *et al*. 2008; Woo & Xiao, 2012); others, however, suggested β_1_AR blockade combined with β_2_AR–G_i_ activation as the preferred strategy for heart failure therapy with particularly striking results in a model of Takotsubo cardiomyopathy (Siedlecka *et al*. 2008, clenbuterol as β_2_AR–G_i_ biased β_2_‐agonist; Paur *et al*. 2012). Takotsubo cardiomyopathy is characterized by ballooning and contractile dysfunction only of the apical portions of the heart in response to excessive emotional stress and subsequent exposure to high levels of catecholamines. Prevention of adrenaline‐mediated G_i_ effects increased mortality, thus providing strong evidence for the beneficial effects of G_i_ coupling in activating β_2_ARs. β_2_AR–G_i_ signalling may thus be essential to counteract hyperactivated β_1_AR–G_s_ signalling (Gorelik *et al*. 2013).

Finally, the general view of the β_1_AR as the ‘bad’ and the β_2_AR as the ‘good’ receptor in heart failure also has been challenged by the finding that deletion of β_2_AR was cardioprotective in a model of genetic cardiomyopathy. Deletion of β_1_AR in this particular mouse mutant was proposed to worsen the phenotype via a PKA‐independent pathway employing Epac (Fajardo *et al*. 2013; Zhang *et al*. 2013). In summary, even though chronic β_1_AR signalling is generally thought to be cardiotoxic and chronic β_2_AR signalling cardioprotective, the outcome of β_1_AR *vs*. β_2_AR activation depends at least partially on the underlying disease type.

## Strategies in heart failure that target βAR signalling

While pharmacological stimulation of βARs is commonly used to stabilize a failing heart in an acute situation, blockage of βARs in chronic heart failure turned out to be beneficial due to disruption of the vicious circle between sympathetic overdrive and maladaptive remodelling processes. Unlike initial expectations from negative inotropic drugs, antagonists of βARs (β‐blockers) improve patients’ symptoms and significantly promote survival when applied carefully at slowly increasing dosages. Multiple studies within the last two decades have shown that β‐blockers improve survival for chronic heart failure by up to 30% (Packer *et al*. 1996a,b; Lechat *et al*. 1998). Low dosages of β‐blockers are sufficient to protect from sympathetic overdrive, thereby preventing β_1_AR‐mediated remodelling processes and restoring β‐adrenergic function by re‐sensitization and increased expression of βARs (Felker, 2001; Lompré *et al*. 2010; Tacon *et al*. 2012; Ponikowski *et al*. 2016). However, not all patients tolerate β‐blockers well and the withdrawal rate is high due to side effects like fatigue, sleep disturbance, depression, weight gain, pulmonary side effects and sexual dysfunction (Packer *et al*. 1996a,b). In heart failure, depression of cardiac contractility further hampers the use of β‐blockers in general or at least at the desired dose. The ideal drug in the treatment of heart failure would increase cardiac output and thereby instantly alleviate symptoms, but without the adverse effects of chronic βAR stimulation.

Several new experimental strategies have been added in recent years to increase cardiac contractility in heart failure by activation of βAR or modulation of βAR downstream signalling, particularly by targeting regulators of myocyte Ca^2+^ cycling. Most attempts to reconstitute *βAR signalling* failed, because they accelerated rather than attenuated deterioration of cardiac morphology and function. These studies evaluated the use of isoproterenol or dobutamine; the inhibition of phosphodiesterase III (PDEIII), an enzyme that degrades cAMP; activation of PKA; inhibition of protein phosphatase 1, an enzyme that reduces PKA‐mediated activation of calcium cycling proteins; or activation of CaMKII (El‐Armouche *et al*. 2008; Lehnart *et al*. 2009; Lompré *et al*. 2010; Tacon *et al*. 2012; Bers, 2014; Ponikowski *et al*. 2016).

Studies aiming at myocyte *Ca^2+^ cycling* yielded more promising results for the treatment of heart failure. Ca^2+^ coordinates myofilament activity in the contractile apparatus of the cardiac myocyte. Upon electrical stimulation, the concentration of Ca^2+^ in the contractile units increases at least 10‐fold, thereby inducing the formation of cross‐bridges between myofilaments. The subsequent conformational changes of the myosin head finally lead to myocardial contraction. Therefore, levels of cellular Ca^2+^ directly correlate with the heart's mechanical function and enhancing myocyte Ca^2+^ cycling increases mechanical force of the contractile units and the rate of contraction and relaxation. Potential therapeutic strategies were evaluated in animal models aiming at nodal points of the signalling cascade such as sarco‐/endoplasmatic reticulum Ca^2+^‐ATPase (SERCA2a), which plays an important role in diastolic Ca^2+^ removal. SERCA2a activity was modulated via deletion of the SERCA2a inhibitor PLN, overexpression of SERCA2a or overexpression of S100 calcium binding protein A1 (S100A1). Further, the LTCC was targeted using the Gβγ scavenger C‐terminus of the β‐adrenergic receptor kinase (βARKct), which leads to disinhibition of G‐protein (Gβγ)‐mediated inhibition of the channel (Slack *et al*. 2001; Schmitt *et al*. 2009; Pleger *et al*. 2011; Völkers *et al*. 2011; Kairouz *et al*. 2012). In healthy hearts, none of these strategies led to rapid deterioration of cardiac function and they all successfully rescued animal models of heart failure. The most‐progressed target, SERCA2a, was evaluated in patients with moderate to severe heart failure in the Calcium Upregulation by Percutaneous Administration of Gene Therapy in Cardiac Disease (CUPID) trial. In phase 1/2, intracoronary infusion of a recombinant adeno‐associated virus (AAV) vector for delivery of SERCA2a DNA appeared promising; however, in a follow‐up study that evaluated the effects on hospitalization and mortality, SERCA2a gene transfer turned out to be safe but did not improve the endpoints. Technical issues leading to inefficient cellular uptake of the viral vector are discussed as likely causes for the failure of the phase 2b CUPID trial. Further investigation of this trial is needed to avoid failure of future gene therapy trials (Pleger *et al*. 2014; Greenberg *et al*. 2014, 2016; Greenberg, 2015; Lother & Hein, 2016).

The underlying reasons why reconstitution of βAR signalling is particularly prone to cardiac damage but reconstitution of Ca^2+^ cycling is rather well‐tolerated or even protective are not yet understood. Remarkably, overexpression of adenylyl cyclase 6 (AC6) safely increased left ventricular function beyond standard heart failure therapy in a recently published phase 1/2 trial of AC6 gene transfer in heart failure patients (Pleger *et al*. 2014; Hammond *et al*. 2016). Unlike other AC subtypes, AC6 has no effect on basal cAMP levels and is only responsive to βAR stimulation suggesting that selective and non‐constitutive activation of βAR downstream targets may be crucial in distinguishing well‐tolerated from detrimental positive inotropy. AC6 is also thought to improve cardiac performance via cAMP‐independent mechanisms that still need to be elucidated (Gao *et al*. 2002; Tang *et al*. 2012).

Recently, RKIP was suggested as a promising strategy to stimulate cardiac contractility and to reconstitute βAR signalling of failing hearts by chronic β_1_AR activation without triggering adverse effects. Unlike AC6, RKIP enhances adrenergic signalling in cardiomyocytes at a different level. RKIP attenuates GRK2 activity and thereby produces a balanced activation of β_1_ARs and β_2_ARs. The following discussion will summarize the potential benefits of this differential activation in failing cardiomyocytes and evaluate RKIP as a therapeutic agent against heart failure. We will further discuss the effects of RKIP on key components of downstream βAR signalling, particularly myocyte Ca^2+^ kinetics, diastolic Ca^2+^ leak and myofilament Ca^2+^ sensitivity since they show characteristic alterations in failing hearts that lead to contractile dysfunction and arrhythmia.

## RKIP – a governor of intracellular signalling

RKIP belongs to the evolutionarily conserved phosphatidylethanolamine‐binding protein (PEBP) family, which has been characterized as a modulator of signal transduction cascades in mammalian cells and has been reviewed in detail by Trakul & Rosner (2005), Granovski & Rosner (2008) and Lorenz *et al*. (2014a). PEBP/RKIP proteins possess a central β‐sheet surrounded by smaller β‐strands and two carboxy‐terminal α‐helices. These structural elements are connected by loops of variable length. Characteristic for this family is a cavity at the surface that consists of dynamically arranged amino acid residues and displays high affinity for small anionic groups such as phosphates, phospholipids and nucleotides (Hengst, 2000; Granovski & Rosner, 2008; Granovski *et al*. 2009). This cavity is also implicated in the binding of RKIP to the kinase Raf‐1 to the extent that reduced flexibility of the cavity favours Raf binding (Granovski *et al*. 2009). Raf‐1 is a member of the Raf–mitogen‐activated protein kinase kinase (MEK)–extracellular signal‐regulated protein kinases 1 and 2 (ERK1/2) cascade that is involved in differentiation, proliferation, cell survival and hypertrophy. RKIP has been shown to inhibit Raf‐1 signalling (Yeung *et al*. 1999), but the mechanism is not yet entirely clarified. While overexpressed RKIP has been postulated to interfere with the interaction of Raf‐1 with its substrate, MEK, endogenous RKIP rather interferes with Raf‐1 activation (Trakul & Rosner, 2005). Phosphorylation of RKIP by protein PKC at serine 153 mediates the release of Raf‐1 from RKIP (Corbit *et al*. 2003; Lorenz *et al*. 2003; Deiss *et al*. 2012). Interestingly, serine 153 phosphorylation triggers an additional mechanistic/structural feature with impact on the control of RKIP interaction partners: it induces RKIP dimerization. A loop structure at the surface of RKIP and in immediate proximity to the PKC phosphorylation site was identified as a part of the dimerization interface (Deiss *et al*. 2012). RKIP dimerization facilitates the release of Raf‐1 but also participates in the substrate switch of RKIP from Raf‐1 to GRK2 since inhibition of RKIP dimerization prevented RKIP/GRK2 binding and, *vice versa*, a dimeric RKIP mutant was able to bind GRK2 in the absence of RKIP^Ser153^ phosphorylation (Deiss *et al*. 2012). As mentioned above, GRK2 is a kinase that phosphorylates activated G‐protein‐coupled receptors (GPCRs), thereby initiating their desensitization and internalization and subsequently blunting receptor signalling (Pierce *et al*. 2002). In mammalian cells, GRK2 is a major feedback inhibitor of GPCRs and has been implicated in diseases such as immune diseases or heart failure. RKIP does not inhibit the catalytic activity of GRK2 but interferes with the GRK2–receptor interaction via its binding to the N‐terminus of GRK2, a part of GRK2 that is important for GRK–receptor interaction (Lorenz *et al*. 2003). This inhibitory mechanism of RKIP enables a largely specific interference of RKIP with GRK2 towards receptor substrates while cytosolic substrates of GRK2 are not affected (Schmid *et al*. 2015). GRK2–RKIP interaction prevents GPCR internalization leading to enhanced GPCR signalling, which, in the heart, enhances contraction and relaxation.

Even though other kinase signalling cascades such as the nuclear factor κ‐light‐chain‐enhancer of activated B‐cells (NFκB) and glycogen synthase kinase‐3β are also known to be regulated by RKIP in cultured cells, thus far only Raf‐1, MEK1 and ERK2 as well as GRK2 have been identified as direct interaction partners of RKIP of which only Raf‐1 and GRK2 have been validated under endogenous conditions (Yeung *et al*. 2001; Lorenz *et al*. 2009, 2014b). In line with its influence on several kinase signalling cascades, RKIP impacts on diverse physiological processes including cell transformation, cell cycle, inflammation, metastasis and cardiomyocyte contractility (Granovsky & Rosner, 2008; Lorenz *et al*. 2014a; Brietz *et al*. 2016). Deletion or downregulation of RKIP resulted for example in deterioration of metastatic cancer, Alzheimer's disease, pulmonary hypertension and heart failure and increased replication of the Newcastle disease virus (Lorenz *et al*. 2014a; Schmid *et al*. 2015; Yin *et al*. 2016).

## RKIP and its function in the heart

**Figure 2:**
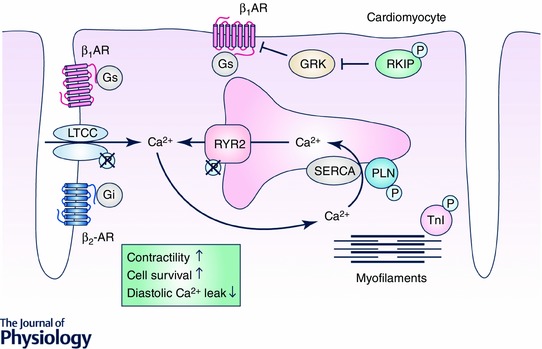
RKIP induces positive inotropy and protects from cell death and diastolic Ca^2+^ leak The Raf kinase inhibitor protein (RKIP) binds GRK2 and inhibits G‐protein‐coupled receptor kinase (GRK)‐mediated receptor phosphorylation, which prevents receptor desensitization and internalization and, thus, increases β‐adrenergic receptor signalling. RKIP increases contractility and relaxation of cardiomyocytes via activated β_1_‐adrenergic receptor (β_1_AR) coupled to stimulatory G‐proteins (G_s_): phosphorylated phospholamban (PLN) dissociates from sarco‐/endoplasmatic reticulum Ca^2+^‐ATPase (SERCA2a) and thereby increases SERCA2a activity, Ca^2+^ loading of the sarcoplasmatic reticulum and cardiomyocyte contractility. Phosphorylation of troponin I (TnI) decreases Ca^2+^ sensivity and thereby increases cardiomyocyte relaxation. RKIP mediates anti‐apoptotic, anti‐fibrotic and anti‐arrhythmic effects via increased β_2_‐adrenergic receptor (β_2_AR) signalling. Continuous signalling of β_2_AR coupled to inhibitory G‐proteins (G_i_) prevents β_1_AR‐stimulated increases in ryanodine receptor 2 (RyR2) and L‐type Ca^2+^ channel (LTCC) phosphorylation and protects from diastolic Ca^2+^ leak.

Cardiac RKIP expression is up‐regulated in heart failure patients and in mice with pressure overload‐induced heart failure, which implies that RKIP is part of the physiological response to stress in cardiac diseases. Indeed, mice with cardiac overexpression of RKIP are protected from heart failure induced by chronic pressure overload (induced by transverse aortic constriction) while RKIP deficiency exaggerated heart failure under these conditions. AAV9‐mediated gene transfer protected wild‐type and RKIP knockout mice from transverse aortic constriction‐induced heart failure (Schmid *et al*. 2015). Recent findings strongly suggest that RKIP provides a new and well‐tolerated mode of sustained βAR activation in the heart by differential stimulation of protective *vs*. detrimental β‐adrenergic signalling (Schmid *et al*. 2015; Fig. 2). The data indicate that the effects of RKIP in the heart are characterized by the following qualities:

(1) RKIP stimulates β_1_AR–G_s_ signalling, which results in enhanced contraction and relaxation via increased PLN and TnI phosphorylation and subsequently increased SERCA2a activity, higher SR Ca^2+^ load and decreased Ca^2+^ sensitivity of myofilaments. Cardiac contractility of RKIP‐overexpressing mice was improved compared to control animals up to an age of at least 12–14 months; and lifespan of RKIP‐overexpressing mice under these conditions was at least as long as of non‐transgenic mice (Schmid *et al*. 2015).
(2) Despite enhanced β_1_AR–G_s_ signalling, RKIP‐stimulated hearts are still able to respond adequately to physiological stress situations because the size of the dobutamine response of RKIP‐overexpressing and wild‐type hearts is similar. This moderate or submaximal activation may play an important role for the observed reduction of cardiomyocyte apoptosis, interstitial fibrosis, brain natriuretic peptide and collagen expression in RKIP‐overexpressing mice compared to wild‐type controls and the overall well‐tolerated positive inotropic phenotype of RKIP‐overexpressing mice (Schmid *et al*. 2015).
(3) Besides β_1_AR–G_s_‐signalling that stimulates the activity of both PKA and CaMKII, RKIP activates β_2_AR–G_i_ in mouse hearts. The simultaneous activation of β_2_AR–G_i_ within the transverse (t)‐tubular region prevents the adverse effects of mere β_1_AR–G_s_, such as diastolic Ca^2+^ leak and cardiac arrhythmia due to hyperphosphorylation and subsequent activation of the RyR2 or hyperphosphorylation of the LTCC. These RKIP effects appear to be mediated by β_2_AR–G_i_ signalling since this protection is absent in RKIP‐overexpressing mice lacking β_2_ARs and in the presence of the G_i_ inhibitor pertussis toxin (Communal *et al*. 1999; Xiao *et al*. 1999; Lehnart *et al*. 2009; Eschenhagen, 2010; Nikolaev *et al*. 2010; Bers, 2014; Schmid *et al*. 2015). Of note, these experiments show that RKIP predominantly activates β_2_AR coupled to G_i_ in mouse hearts, even though RKIP in principle is capable of activating β_2_AR–G_s_ as demonstrated in cell cultures (Lorenz *et al*. 2003). Further evidence for a central role of β_2_AR on the protective effects of RKIP in the heart is the absence of protection from cardiac remodelling, i.e. apoptosis and interstitial fibrosis, in RKIP transgenic mice lacking the β_2_AR as well as a reduced overall survival compared to β_2_KO controls – effects that are reported to result from mere β_1_AR–G_s_ signalling (Schmid *et al*. 2015). The switch of β_2_AR from G_s_ to G_i_ in RKIP transgenic mice seemed to be due to the enduring β_1_AR–G_s_–PKA activation as indicated by the characteristic phosphorylation patterns of βAR downstream targets (Daaka *et al*. 1997; Xiao *et al*. 1999). Remarkably, overall β_2_AR phosphorylation is significantly reduced in RKIP transgenic mice, consistent with GRK2 inhibition and absence of β_2_AR desensitization in RKIP transgenic mice (Rockmann *et al*. 2002; Houslay & Baillie, 2005; Schmid *et al*. 2015).
(4) RKIP was found to inhibit βAR downregulation and thereby secures sustained positive inotropy, which is not achieved by direct agonist‐mediated βAR stimulation (Lorenz *et al*. 2003; Schmid *et al*. 2015) (Figs  1 and 2).
(5) RKIP promotes cell survival. RKIP overexpression reduced cardiomyocyte apoptosis, whereas deletion of RKIP (RKIP^−/−^) dramatically increased it. This effect is β_2_AR dependent since the protection from apoptosis is absent in RKIP‐transgenic mice lacking the β_2_AR. Interestingly, β_2_AR–G_i_ is known to stimulate the kinase Akt, which in turn mediates anti‐apoptotic effects (Chesley *et al*. 2000; Talan *et al*. 2011). In line with β_2_AR–G_i_ activation by RKIP, Akt activation was enhanced in RKIP‐overexpressing mice and was dependent on β_2_AR and pertussis toxin‐sensitive G_i_ proteins. These findings suggest that RKIP mediates cell survival via Akt.

However, as described above, RKIP not only increases GPCR signalling via GRK inhibition, but also inhibits mitogen‐activated protein kinase signalling dependent on its phosphorylation status: RKIP acts as a GRK inhibitor in its PKC phosphorylated form (pRKIP^Ser153^), but in the absence of Ser153 phosphorylation it acts as a Raf‐1 inhibitor. In the heart, Raf–MEK–ERK1/2 signalling promotes cell survival (Punn *et al*. 2000; Harris *et al*. 2004; Heineke & Molkentin, 2006; Purcell *et al*. 2007; Sheikh *et al*. 2008; Cheng *et al*. 2011; Van Berlo *et al*. 2011). Thus, in its unphosphorylated form, RKIP could potentially increase cardiomyocyte death. However, RKIP in the heart mainly exists in its phosphorylated form, so that the Raf‐1 inhibitory effect of RKIP is absent in the heart. Even moderate RKIP overexpression of up to 8‐fold revealed no inhibitory effect of RKIP on Raf‐1/MEK/ERK1/2. Potential side effects of this new cardioprotective strategy may occur at very high overexpression levels of RKIP that exceed the ability of PKC to fully phosphorylate RKIP and may result in RKIP‐mediated Raf/MEK/ERK1/2 inhibition and increased apoptosis associated with signs of heart failure as seen by Fu *et al*. or by cardiac overexpression of a phosphorylation‐deficient mutant of RKIP, RKIP^S153A^ (Bueno *et al*. 2000; Lorenz *et al*. 2003; Fu *et al*. 2013; Ruppert *et al*. 2013; Schmid *et al*. 2015).

Compared to other positive inotropic strategies in heart failure therapy, the biochemical and phenotypic effects of RKIP substantiate the hypothesis that a successful positive inotropic strategy should not induce an unselective activation of βAR downstream targets (as for βAR agonists, PDEIII inhibitors or β_1_AR‐overexpressing mice) but rather circumvent activation of the RyR2 (as in PLN^–/–^ mice, SERCA2a or βARKct overexpression) or even protect from RyR2 sensitization (as in GRK2^–/–^ mice, S100A1 and RKIP transgenic mice) (Kairouz *et al*. 2012; Respress *et al*. 2012; Bers, 2014; Pleger *et al*. 2014; Ritterhoff *et al*. 2015). RKIP achieves this well‐tolerated βAR stimulation with positive inotropy and lusitropy in RKIP‐overexpressing mice by concomitant activation of β_2_ARs (in their G_i_‐coupled mode) that counteracts several maladaptive β_1_AR effects such as RyR2 sensitization, diastolic Ca^2+^ leaks and arrhythmia as well as apoptosis and fibrosis. Indeed, ‘sole’ β_1_AR activation as provided in RKIP transgenic mice lacking the β_2_AR (β_2_KO) increased cardiac contractility, but also reduced overall survival of ageing RKIP transgenic mice (RKIP/β_2_KO) compared to β_2_KO controls (Schmid *et al*. 2015). Taken together, the consequences of enhanced βAR signalling in the heart appear to be highly dependent on the type and the extent of activated signalling elements.

In sum, RKIP differentially modulates several molecular events downstream of βAR, which appears promising for heart failure therapy. In the following, we will discuss the differential regulation of βAR receptors by RKIP, its effects on myocyte Ca^2+^ kinetics and distribution and how failing hearts may benefit from these alterations.

## Depressed myocyte Ca^2+^ cycling and heart disease

Ca^2+^ enters the cardiomyocyte via the LTCCs, which are predominantly located within the t‐tubuli of the sarcolemma in close neighbourhood to the sarcoplasmic Ca^2+^ release channels, RyR2. These functional dyads facilitate the rapid increase of cytosolic Ca^2+^ levels upon depolarization of the cell leading to myofilament contraction. Cardiac relaxation is initiated by Ca^2+^ removal from the cytosol. In human myocytes, 74% of diastolic Ca^2+^ removal is accomplished by the Ca^2+^‐ATPase SERCA2a, 24% by the Na^+^/Ca^2+^ exchanger (NCX), 1% by the sarcolemmal Ca^2+^‐ATPase (PMCA) and 1% by the mitochondrial Ca^2+^ uniporter) (Bers, 2014). Since SERCA2a eliminates the largest share (even 93% of Ca^2+^ removal in myocytes from mice and rats), the SERCA2a regulator PLN plays a pivotal role in modulating myocyte Ca^2+^ distribution and kinetics. PLN inhibits SERCA2a, thereby attenuating the rate of Ca^2+^ transport to the SR. Phosphorylation of PLN by PKA at serine 16 can almost fully relieve this inhibitory effect leading to a pronounced acceleration of SR Ca^2+^ uptake. As Ca^2+^ is sequestered by the SR, in the sarcomeres TnI inhibition of actin–myosin interactions is re‐established and myocytes relax (Rockman *et al*. 2002).

In heart failure, depressed contractility is associated with depressed myocyte Ca^2+^ cycling. Although the causes of heart failure can vary widely, e.g. myocardial infarction, arterial hypertension, infections or genetic defects, the pattern of abnormal Ca^2+^ metabolism is relatively uniform (overview in Lehnart *et al*. 2009). In failing hearts, Ca^2+^ release is typically reduced, consistent with a decrease in contractility and force generation (van der Velden *et al*. 2004; Avner *et al*. 2011; Haghighi *et al*. 2014). Further, SR Ca^2+^ reuptake during diastole is slow and diastolic Ca^2+^ levels are elevated due to a diastolic Ca^2+^ leak (via RyR2), but primarily due to reduced SERCA2a activity. Depression of the Ca^2+^ pump results from reduced SERCA2a expression in failing hearts, whereas PLN levels remain stable leading to a reduced SERCA/PLN ratio (Hasenfuss & Pieske, 2002). SR Ca^2+^ transport is further inhibited by a reduction of PLN phosphorylation, most likely as a result of increased protein phosphatase‐1 activity and downregulation of βAR density (Weber *et al*. 2016).

The identification of inherited mutations in Ca^2+^ regulatory proteins that caused alterations of protein function and induced dilated cardiomyopathy and terminal heart failure finally proved the concept that the Ca^2+^ cycling alterations in failing hearts are not secondary events or bystander in the course of the disease, but play a causative role in myocardial remodelling and the deterioration of cardiac function (Haghighi *et al*. 2003; Schmitt *et al*. 2003). Therefore, restoration of SR Ca^2+^ cycling holds promise for the treatment of heart failure.

RKIP seems to provide a promising approach for the restoration of depressed Ca^2+^ cycling. It increases myocyte Ca^2+^ transients at baseline and also upon bolus application of caffeine indicative of increased Ca^2+^ release. Increased Ca^2+^ release during systole in RKIP‐overexpressing cardiomyocytes is most likely due to accelerated Ca^2+^ reuptake into the SR during diastole, which is mainly due to enhanced PLN phosphorylation at serine 16 (PKA site) and threonine 17 (CaMKII site) leading to efficient release of PLN from SERCA2a and subsequent activation of SERCA2a. The RKIP‐induced increase in Ca^2+^ reuptake during diastole may further be supported by an accelerated Ca^2+^ release from the myofilaments mediated by enhanced TnI phosphorylation at the serine residues 23 and 24. Further, via GRK inhibition, RKIP prevents βAR desensitization and βAR degradation in a heart under chronic sympathetic stress, which subsequently secures efficient and continuous Ca^2+^ cycling. In addition, RKIP overexpression is able to prevent a loss of SERCA2a expression in a failing mouse heart, which in turn also ensures effective Ca^2+^ cycling in cardiomyocytes.

In sum, RKIP improves cardiac performance in healthy hearts and in failing hearts. Since the extent of the contractile response depends on the amount of activating Ca^2+^, the enhanced Ca^2+^ load would explain the hypercontractile phenotype of RKIP‐overexpressing hearts and the improved cardiac function in a mouse model of heart failure due to chronic pressure overload (Schmid *et al*. 2015). RKIP is an elegant example of achieving a stable and physiological (i.e. still regulatable) increase in Ca^2+^ cycling on several molecular levels via restoring expression and function of both βAR and direct regulators of myocyte Ca^2+^ cycling.

## Increased Ca^2+^ sensitivity and heart disease

Ca^2+^ sensitivity of myofilaments affects contraction, relaxation and remodelling of the myocardium as well as cardiac rhythm. Increased Ca^2+^ sensitivity was observed in end‐stage heart failure and in heart tissue 3–4 days after myocardial infarction (van der Velden *et al*. 2004; Avner *et al*. 2011). Further, in hypertrophic cardiomyopathy, the most frequent cause of sudden cardiac death in the young population, an increase of myofilament Ca^2+^ sensitivity has been proposed as a central disease mechanism (Landstrom & Ackerman 2012; Deftereos *et al*. 2016). Increased Ca^2+^ sensitivity is often associated with high susceptibility for ventricular tachycardia and sudden cardiac death. Desensitization of myofilaments to Ca^2+^ was suggested to reduce the risk of arrhythmias by stabilizing action potential generation and propagation, because high Ca^2+^ sensitivity would prolong Ca^2+^ transients and slow down the propagation of action potentials, thereby fostering the generation of electrical re‐entry (Huke & Knollmann, 2010; Tardiff *et al*. 2015). The causative relation to arrhythmia was underlined by human and animal studies that found increased episodes of ventricular tachycardia after myocardial infarction and in heart failure upon treatment with the Ca^2+^ sensitizer levosimendan (Flevari *et al*. 2006). In contrast, the myosin inhibitor blebbistatin reduced myofilament Ca^2+^ sensitivity and prevented ventricular tachycardia in troponin T mutant mice (Baudenbacher *et al*. 2008). These examples demonstrate the broad therapeutic possibilities of Ca^2+^ desensitizing agents to fight arrhythmias and myocardial remodelling as well as contractile dysfunction.

Schmid *et al*. (2015) found RKIP to increase TnI phosphorylation at S23/S24. This PKA‐dependent phosphorylation decreases Ca^2+^ sensitivity of the Tn complex because phosphorylation reduces its Ca^2+^ affinity (Cheng *et al*. 2015). The strong therapeutic potential of decreasing Ca^2+^ sensitivity by TnI phosphorylation was demonstrated by the expression of a pseudo‐phosphorylated TnI mutant that rescued the morphological and functional changes of the heart in an animal model of hypertrophic cardiomyopathy caused by an E180G α‐Tm mutant with increased myofilament Ca^2+^ sensitivity (Alves *et al*. 2014). RKIP appears as a particularly attractive tool for Ca^2+^ desensitization of myofilaments because it also exhibits positive inotropy via enhanced β_1_AR signalling and antiarrhythmic effects via reduced RyR2 and LTCC phosphorylation, and it prevents apoptosis and maladaptive remodelling by Akt stimulation.

## Diastolic Ca^2+^ leak and heart disease

Besides desensitization of myofilaments by increasing TnI phosphorylation, Schmid *et al*. (2015) also showed RKIP to reduce the frequency of Ca^2+^ sparks and Ca^2+^ waves. Ca^2+^ sparks occur if a cluster of RyR2 produces a local Ca^2+^ release from the SR. With every heartbeat, the action potential synchronizes the almost simultaneous opening of thousands of RyR2 clusters within a myocyte leading to a Ca^2+^ transient that initiates contraction. In contrast, the local Ca^2+^ increase caused by a spontaneous Ca^2+^ spark can trigger Ca^2+^ release only from neighbouring RyR2 clusters via a Ca^2+^‐induced Ca^2+^ release. The resulting propagating wave may induce Ca^2+^ elimination by the NCX causing an inward current (1 Ca^2+^ out–3 Na^+^ in) and both early and delayed afterdepolarizations that trigger aberrant electrical activity and arrhythmias of the heart (reviewed in Bers, 2014). The clinical relevance of this pathomechanism was demonstrated by the finding of disease‐causing RyR2 mutations in patients with catecholaminergic polymorphic ventricular tachycardia, because the genetic defects put affected individuals at risk for stress‐induced ventricular tachycardia (Priori & Chen, 2011). Not only do Ca^2+^ sparks trigger arrhythmia, but the diastolic loss of Ca^2+^ also reduces SR Ca^2+^ content. As a consequence of this loss, the systolic Ca^2+^‐induced Ca^2+^ release is smaller leading to reduced contraction of the heart. The Ca^2+^ leak also impairs myocardial relaxation, because it slows down cytosolic Ca^2+^ clearance during diastole of the heart and it may cause diastolic activation of contractile proteins.

RKIP reduces the frequency of Ca^2+^ sparks and Ca^2+^ waves by reducing phosphorylation of RyR2 at SS2808/2814 (Schmid *et al*. 2015). Hyperphosphorylation of RyR2 is known to induce diastolic SR Ca^2+^ leakage, which predisposes for arrhythmias (Bers, 2014). The respective role of S2808 and S2814 phosphorylation by PKA or CaMKII in this scenario, however, is controversial in the field (Eschenhagen, 2010; Bers, 2014). The reduction of RyR2 phosphorylation by RKIP despite enhancing β‐adrenergic signalling is explained by β_2_AR–G_i_‐coupled signalling induced by RKIP (Schmid *et al*. 2015). Since β_2_ARs are believed to be concentrated within the t‐tubular region of the cardiomyocyte (Kuschel *et al*. 1999; Orchard & Brette, 2007; Nikolaev *et al*. 2010), β_2_AR–G_i_ signalling within the t‐tubular region seems to protect βAR targets in close proximity to β_2_ARs such as the RyR2 and LTCC from hypersensitization. It is noteworthy that the resulting stabilization of RyR2 is strong enough to reduce the incidence of Ca^2+^ sparks despite increased SR Ca^2+^ contents, a condition that can trigger spontaneous Ca^2+^ release.

Pharmacological and gene therapeutic studies are aimed at fixing the Ca^2+^ leak, increasing the Ca^2+^ transient and enhancing cytosolic Ca^2+^ clearance (reviewed in Marks, 2013). RKIP is a positive inotropic strategy that circumvents RyR2 sensitization and seems to fulfil all of these requirements. RKIP (1) reduces the occurrence of Ca^2+^ sparks by β_2_AR‐mediated stabilization of RyR2, (2) accelerates cytosolic Ca^2+^ elimination by PKA‐dependent phosphorylation of PLN and (3) increases SR Ca^2+^ filling and therefore the size of the Ca^2+^ transients. Finally, RKIP overexpression prevented morphological and functional maladaptation of the heart in mice subjected to long‐term pressure overload. Future studies will show if these beneficial effects hold true for the rescue of independent models of heart failure.

## Conclusion

RKIP is an endogenous protein that exhibits a combination of favourable effects for heart failure patients: (1) the gain/increase of cardiac contractile efficiency by the activation of G_s_ signalling/β_1_AR leading to functional recovery of the heart and (2) the protection of the heart under sympathetic stress from exaggerated β_1_AR downstream signalling including protection from apoptosis and pro‐arrhythmic adverse effects via β_2_AR activation. This approach promises a new therapeutic strategy to achieve well‐tolerated long‐term increases in cardiac contractility. RKIP comprises several favourable characteristic effects on calcium cycling, calcium sensitivity, G‐protein recruitment to βARs and a physiological extent or range of βAR activation and has proven protective in murine heart failure. Future studies will further unravel the signalling network induced by RKIP that is responsible for the well‐tolerated mode of βAR activation and evaluate its therapeutic efficacy in various disease entities.

## Additional information

### Competing interests

The authors have no competing interests.

### Author contributions

All authors have approved the final version of the manuscript and agreed to be accountable for all aspects of the work. All persons designated as authors qualify for authorship, and all those who qualify for authorship are listed.

### Funding

This work was supported by Deutsche Forschungsgemeinschaft (DFG; Sonderforschungsbereich SFB688 (TPA17 to K.L., Fonds 881067) and SFB 1116 (TPA02 to J.P.S.)), by Bundesministerium für Bildung und Forschung (BMBF; Comprehensive Heart Failure Centre Würzburg; project MY1 to K.L., 98519102 (8502/0)) and by the Ministry for Innovation, Science and Research of the Federal State of North Rhine‐Westphalia (K.L.). A PhD position was awarded to T.B. by the Elite Network of Bavaria within the IDK ‘receptor dynamics’.
